# Tapping for Ascites

**Published:** 1889-02

**Authors:** Benjamin W. Richardson

**Affiliations:** London, England


### TAPPING FOR ASCITES.

BY BENJAMIN W. RICHARDSON, M. D.,

London, England.

In tapping for ascites I have long practiced a little plan which saves a great deal of trouble, and without any extra apparatus entirely prevents the entrance of air into the abdominal cavity. I slip over the canula a piece of india-rubber tubing, and turn the surface of the tube over the end so as to make a flat surface of rubber. I then push the trocar through the rubber, and make it go right home. There is no difficulty in the process whatever, and no interference with the entrance of the trocar through the tissues when the puncture is made. On withdrawing the trocar the opening through the soft rubber closes of itself, and the escaping fluid runs readily through the length of the tube into any utensil that may be placed to receive it.

In my early days of practice the almost universal custom was to use a large trocar and to draw off the fluid as rapidly as possible. Under this plan it was necessary to make provision for compressing the abdomen by the bandage, as the body quickly collapsed, in order to prevent the occurrence of syncope from sudden collapse. The practice was bad, and for many years I have substituted for it the employment of a small trocar, only introducing a large one if the fluid that may flow be too thick and glutinous to pass through a tube of narrow caliber. With a small tube conveying a stream of not more than the eighth part of an inch in diameter, the evacuation of the cavity is slow; but the operation is unattended with any sign of faintness except from fear or mental nervousness on the part of the patient.

To prevent pain in the operation I always use ether spray for benumbing the surface at the point of puncture. It is well to freeze over a space of a disc an inch in diameter, and when the part played on by the spray is quite frozen to put the point of the warm finger on the spot in the centre of the disc, where the puncture is about to be made, so as to soften the frozen skin. This does not restore the sensibility if there be left plenty of frozen tissue around. I have used the spray in this manner thirty times, for tapping, have never failed in rendering the operation perfectly painless, and have never had any untowered result. One patient whom I tapped seven times, and who become accustomed to look on the proceeding with the most perfect unconcern, told me that she was at no time conscious of any sensation beyond a slight jerk which was not at all painful. It may therefore be supposed that the peritoneum, as well as the skin, is rendered insensible to the pain of the puncture. In freezing with the spray it is best to proceed slowly, devoting five minutes to the process, so as to insure deep local anæsthesia.—*Col. & C. Rec.*

